# 

**DOI:** 10.1192/bjb.2025.16

**Published:** 2026-02

**Authors:** Savva Pronin

**Affiliations:** Specialty Trainee in Child and Adolescent Psychiatry, Tavistock and Portman NHS Foundation Trust, London, UK.

**Figure:**
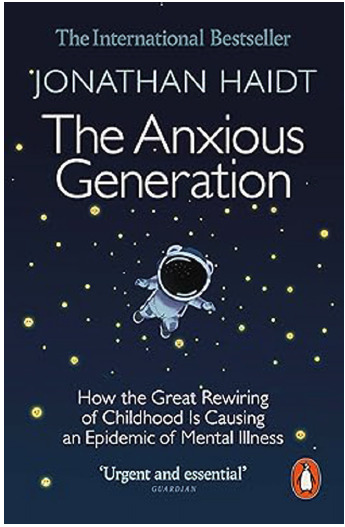

Growing up as a late millennial through the rise of smartphones and social media, I often noticed how those just a few years younger seemed very different from previous generations and suspected that growing up immersed in a digital world may profoundly shape their development. Parodies like *South Park* and stand-up comedians joked about this modern obsessionality but today, in child and adolescent mental health services, we see it daily: young people glued to their phones throughout appointments, afraid of interacting in person, or struggling with screen-induced insomnia. Jonathan Haidt, a social psychologist and author, crystallises these concerns by blending compelling data with thought-provoking narrative in *The Anxious Generation.*

Divided into four sections, the book methodically addresses: mental health trends in adolescents since 2010, the changing nature of childhood, the detrimental impact of a phone-based upbringing and potential interventions to reverse this worrying trajectory. He hones in on 2010 because of what he terms ‘The Great Rewiring’, which can be summarised as ‘underprotection online and overprotection in reality’. The writing is accessible and engaging, enhanced by metaphors, images, graphs, real-life examples and diverse sources – from Instagram posts and hacker leaks to research papers – yet also giving space to the voices of young people. He includes an extended notes section, thorough references and an online supplement for further reading. The breadth of his inquiry is impressive, encompassing a variety of disciplines, such as attachment theory, neuroscience, history, anthropology and politics – I am sure most readers would find something new to learn. Haidt’s passion for the subject is palpable, as is his sense of urgency.

He tackles the complex topic of causation in social science but provides the data for you to reach your own conclusions and challenge alternative hypotheses. Although most discussion purposefully pre-dates the COVID-19 pandemic, which has it own separate mental health consequences and changed the world even further since, the observations remain timely, particularly with the imminent impact of widespread artificial intelligence. To wait for long-term outcome data may result in lost opportunity in the present. Although he may paint a bleak picture, Haidt offers hope through practical suggestions, aiming to inspire awareness and activism at individual, societal and governmental levels.

For parents, clinicians, policymakers, educators and anyone concerned with the mental health of young people, *The Anxious Generation* is a beneficial read. It provides not only a guide to understanding this critical moment in modern history but also a call to action for creating a healthier and more adaptive future. Smartphones are doubtlessly here to stay and how we adapt to them is important; hopefully, we can keep the negative effects confined to as few generations as possible.

